# Dietary Methionine Restriction Improves Gut Health and Alters the Plasma Metabolomic Profile in Rats by Modulating the Composition of the Gut Microbiota

**DOI:** 10.3390/ijms25073657

**Published:** 2024-03-25

**Authors:** Mei Yang, Qian Xie, Yintao Xiao, Minglong Xia, Jiashun Chen, Bi-E Tan, Yulong Yin

**Affiliations:** 1 Key Laboratory of Hunan Province for the Products Quality Regulation of Livestock and Poultry, College of Animal Science and Technology, Hunan Agricultural University, Changsha 410128, China; aym1223@163.com (M.Y.);; 2 Yuelushan Laboratory, Changsha 410128, China; 3 Key Laboratory of Agro-Ecological Processes in Subtropical Region, National Engineering Laboratory for Pollution Control and Waste Utilization in Livestock and Poultry Production, Institute of Subtropical Agriculture, Chinese Academy of Sciences, Changsha 410125, China

**Keywords:** methionine restriction, intestinal barrier, *ZO-1*, SCFAs, BAs

## Abstract

Dietary methionine restriction (MetR) offers an integrated set of beneficial health effects, including delaying aging, extending health span, preventing fat accumulation, and reducing oxidative stress. This study aimed to investigate whether MetR exerts entero-protective effects by modulating intestinal flora, and the effect of MetR on plasma metabolites in rats. Rats were fed diets containing 0.86% methionine (CON group) and 0.17% methionine (MetR group) for 6 weeks. Several indicators of inflammation, gut microbiota, plasma metabolites, and intestinal barrier function were measured. 16S rRNA gene sequencing was used to analyze the cecal microbiota. The MetR diet reduced the plasma and colonic inflammatory factor levels. The MetR diet significantly improved intestinal barrier function by increasing the mRNA expression of tight junction proteins, such as zonula occludens (*ZO*)-1, claudin-3, and claudin-5. In addition, MetR significantly increased the levels of short-chain fatty acids (SCFAs) by increasing the abundance of SCFAs-producing *Erysipclotxichaceae* and *Clostridium_sensu_stricto_1* and decreasing the abundance of pro-inflammatory bacteria *Proteobacteria* and *Escherichia-Shigella*. Furthermore, MetR reduced the plasma levels of taurochenodeoxycholate-7-sulfate, taurocholic acid, and tauro-ursodeoxycholic acid. Correlation analysis identified that colonic acetate, total colonic SCFAs, 8-acetylegelolide, collettiside I, 6-methyladenine, and cholic acid glucuronide showed a significant positive correlation with *Clostridium_sensu_stricto_1* abundance but a significant negative correlation with *Escherichia-Shigella* and *Enterococcus* abundance. MetR improved gut health and altered the plasma metabolic profile by regulating the gut microbiota in rats.

## 1. Introduction

Nutrition exerts profound effects on health, and dietary interventions are commonly used to treat metabolic disorders. Methionine is a sulfur-containing essential amino acid that is required for normal growth and development [1]. Methionine restriction (MetR) is gaining popularity due to its several health benefits, including improved stress resistance, increased longevity, and inhibition of fat deposition in experimental animals [2,3]. More specifically, both 80 and 40% MetR diets are associated with lifespan extension and metabolic health from yeast to rodents, which may be partially mediated through a reduction in oxidative stress [4]. MetR improves hepatic steatosis and delays the development of non-alcoholic steatohepatitis through antioxidant effects and alteration in lipid composition in obese mice [5,6]. In a recent study, MetR reduced liver fat content by regulating fatty acid oxidation and lipid catabolism in high-fat-fed mice [7].

The diverse and dynamic microbial community contributes to maintaining healthy gut homeostasis [8]. The integrity of the intestinal barrier, which is supported by the gut microbiota, is essential for maintaining the stability of the body [9]. Disruption and depletion of the gut microbiota may therefore affect the gut barrier and cause persistent systemic inflammation [10]. Microbiome–host interactions and microbial metabolites are associated with the capacity to produce inflammatory factors [11]. As end products of carbohydrates fermented by the gut microbiota, short-chain fatty acids (SCFAs) are involved in ameliorating host metabolic syndromes [12].

Research has highlighted the role of metabolites in mediating the interaction between the gut microbiota and the host diet. For example, bile acids (BAs) play a critical role in modulating host inflammation and the gut barrier [12,13]. In addition to their well-established role in lipid metabolism and maintaining cholesterol homeostasis, BAs also act as signaling molecules that reshape the composition of the gut microbiota through BA receptors, such as the farnesoid X receptor (FXR), which are involved in entero-protection in order to inhibit bacterial overgrowth and mucosal injury in the ileum [14].

Although previous studies have speculated that MetR exerts beneficial effects on the gut by improving epithelial barrier function in normal rats [15], the role and mechanism of the gut microbiota, and especially its metabolites, on gut health in dietary MetR intervention require further research. Therefore, we hypothesized that MetR plays a role in gut health and investigate whether the effects of MetR on plasma metabolomics are related to the modulation of the gut microbiota composition. The aim of this study was to analyze the effect of MetR diet on gut health in rats using 16S rRNA sequencing combined with plasma metabolomics. We sought to identify plasma metabolites that were associated with the MetR dietary supplements, including taurine-conjugated BAs. We also determined inflammatory cytokines, intestinal tight junction proteins, and SCFAs to investigate the effects of MetR on the plasma metabolome and to identify underlying mechanisms for maintaining gut health. The present study may contribute to the provision of new therapeutic strategies from the perspective of gut microbiota.

## 2. Results

### 2.1. Effects of MetR on Plasma LpS, D-Lactate, DAO, and Cytokine Levels

**Figure 1:**
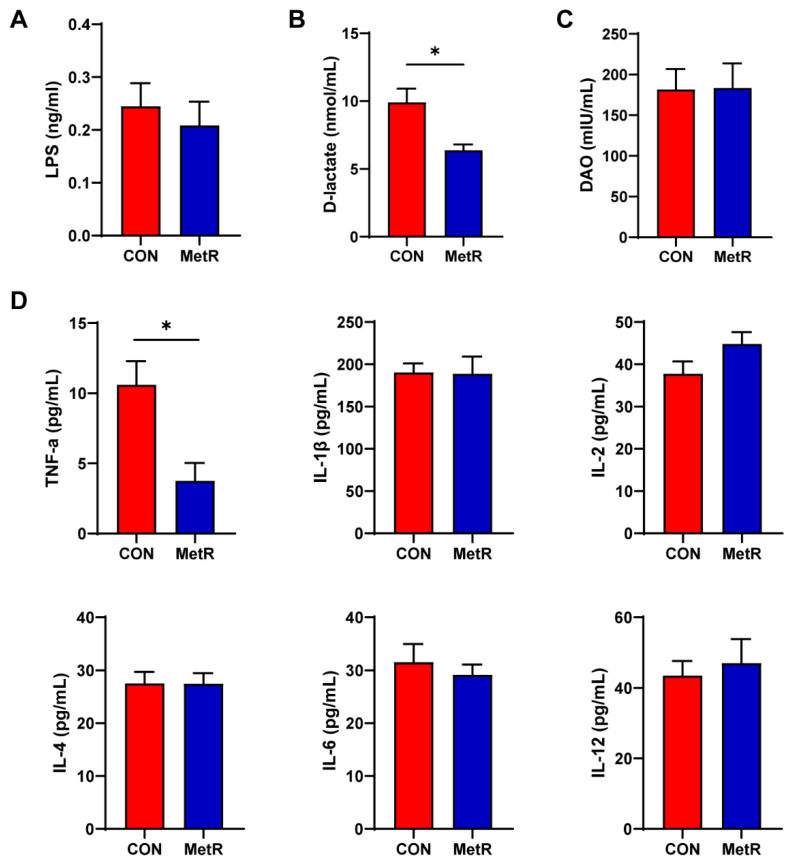
The plasma (**A**) LPS, (**B**) DAO, (**C**) D-lactate, (**D**) TNF-α, IL-1β, IL-2, IL-4, IL-6, and IL-12 levels in the rat. Data are expressed as the mean ± SEM for *n* = 10, * *p* < 0.05 versus CON.

The plasma level of D-lactate was significantly reduced in rats in the MetR group compared to those in the CON group (*p* < 0.05) (Figure 1B). In addition, the LPS and DAO level in the plasma were not significantly different between the CON group and MetR group (*p* > 0.05, Figure 1A,C). Furthermore, MetR significantly decreased the TNF-α level relative to the control rat (*p* < 0.05) (Figure 1D). There were no significant differences in the plasma levels of IL-1β, IL-2, IL-4, IL-6, and IL-12 between the MetR and CON groups (*p* > 0.05, Figure 1D).

### 2.2. Effects of MetR on Colonic Cytokine Levels, Barrier Function, and the Morphological Structure of the Colon

**Figure 2:**
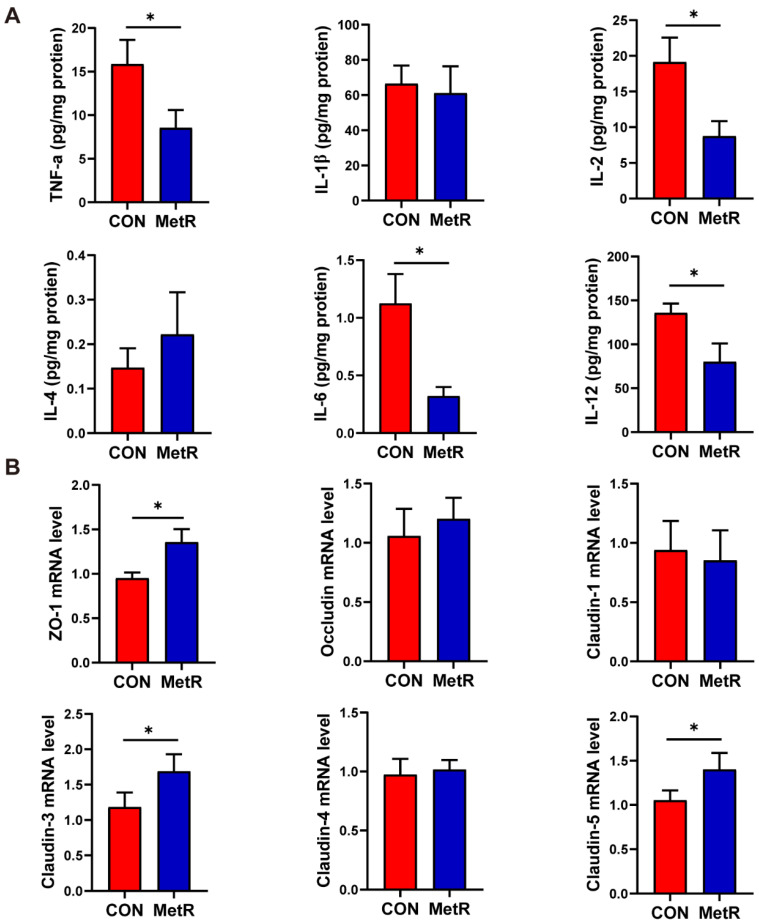
(**A**) Inflammatory factors and (**B**) mRNA expression levels of markers of tight junction proteins on the colon tissue. Data are expressed as the mean ± SEM for *n* = 10, * *p* < 0.05 versus CON.

**Figure 3:**
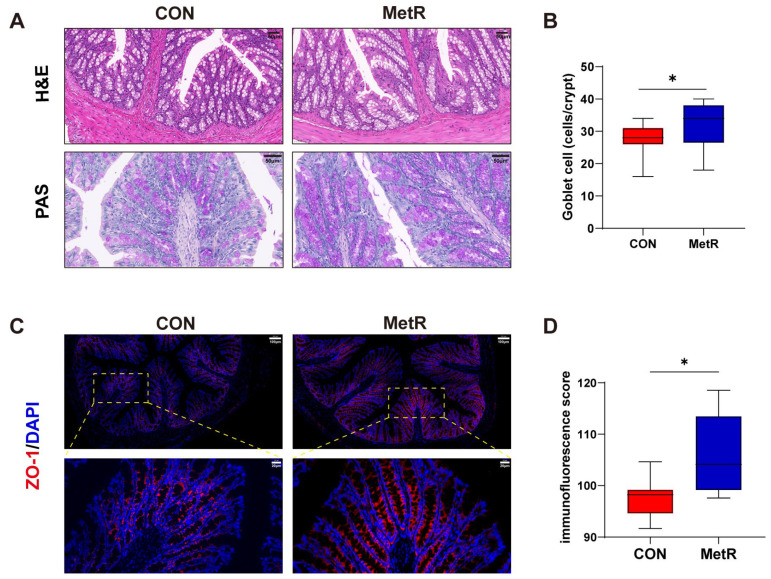
(**A**) Representative images of colon H and E and PAS staining. (**B**) The goblet cell numbers per crypt. (**C**) Representative immunofluorescence images of colonic sections stained with ZO-1 antibody. The dashed rectangle shows the image below with higher magnification. (**D**) Immunofluorescence score. Data are expressed as the mean ± SEM for *n* = 10, * *p* < 0.05 versus CON.

The cytokine levels in the colon are shown in Figure 2A. The levels of TNF-α, IL-2, IL-6, and IL-12 were significantly decreased in the MetR group compared to the CON group (*p* < 0.05). The qRT–PCR analysis revealed an increase in the abundance of zonula occludens (*ZO*)-1, claudin-3, and claudin-5 in colonic samples from rats in the MetR group relative to those in the CON group (*p* < 0.05) (Figure 2B). However, no significant changes in the mRNA expression of occludin and claudin-1 were observed in the colon of rats in the MetR group. Representative photo-micrographs of the H and E-stained and PAS-stained colon tissues are shown in Figure 3A. Goblet cell in MetR group was significantly higher than that in CON group (*p* < 0.05) (Figure 3B). Similarly, MetR significantly upregulated the protein expression of *ZO-1* compared to the CON group (*p* < 0.05) (Figure 3C,D).

### 2.3. Effects of MetR on Cecal Microbiota

**Figure 4:**
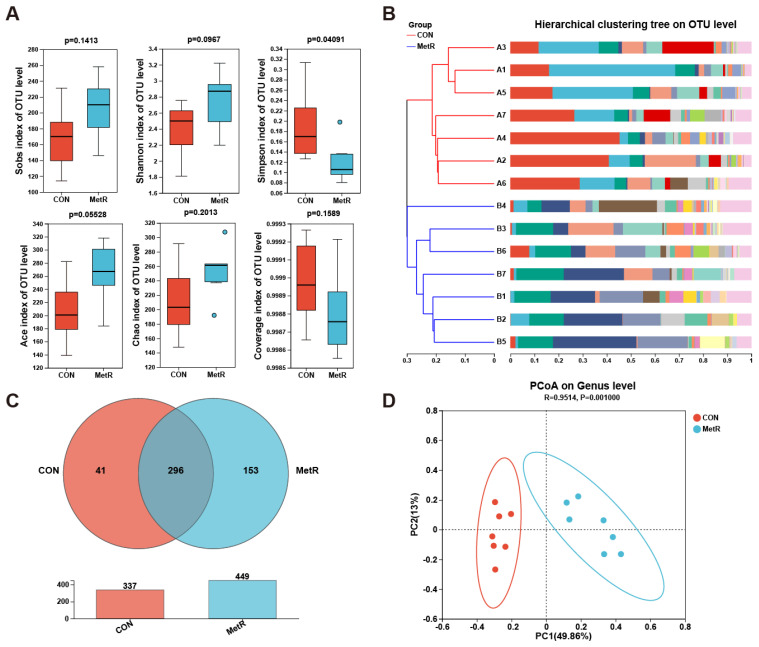
(**A**) Community diversity and richness among CON and MetR group. (**B**) Different colors in the figure represent samples in different groups. The degree of similarity between samples is reflected by the level of aggregation in the graph. (**C**) Venn diagram showing shared and unique OTUs among the two groups. (**D**) PCoA of the bacterial community structure among CON and MetR group. PCoA score plots showed significantly separated clusters between CON and MetR group. Data are expressed as the mean ± SEM for *n* = 7.

The results of the common alpha-diversity analysis (Sob index, Shannon index, Simpson index, Ace index, Chao index, and Coverage index) are shown in Figure 4A. The Simpson index of rats in the MetR group was significantly decreased relative to that of rats in the CON group (*p* < 0.05, Figure 4A). Moreover, MetR administration tended to restore the reduction of the ACE index, but the increase did not reach statistical difference (*p* > 0.05, Figure 4A). We found 296 common OTUs among two groups, while the number of OTUs, about 153, shared by MetR group was higher than that in CON group, with only 41 OTUs (Figure 4C). The Bray-Curtis distance-based PCoA and cluster tree revealed that the CON and MetR group exhibited a distinct clustering of microbiota composition (Figure 4B,D).

**Figure 5:**
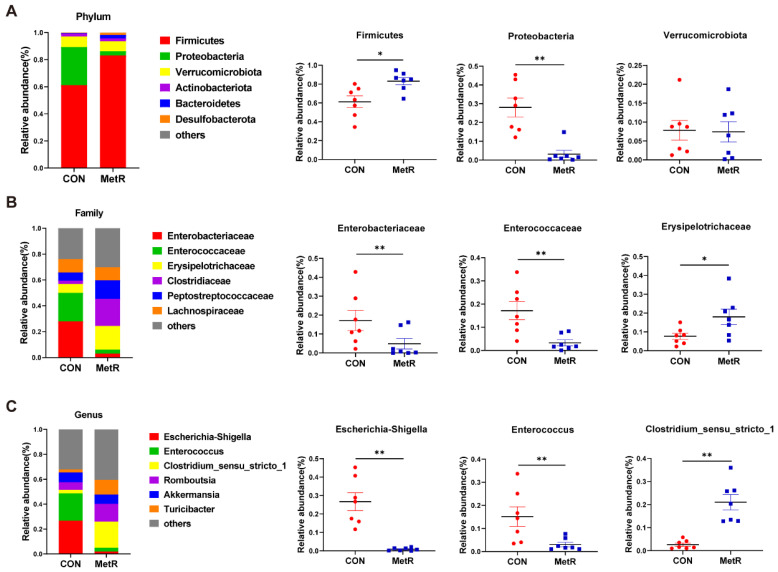
Microbial composition at the (**A**) phylum level, (**B**) family level, and (**C**) genus level. Each bar represents the relative abundance of each bacterial taxa of rats. Data are expressed as the mean ± SEM for *n* = 7, * *p* < 0.05, ** *p* < 0.01 versus CON.

The bacterial sequences of cecal contents from the two experimental groups were examined at the phylum level. Based on the relative abundance of gut microbiota, the top five phyla are displayed (Figure 5A). The phylum level analysis showed that MetR feeding resulted in a significant decrease in the relative abundance of *Proteobacteria* and a significant increase in that of *Firmicutes* (*p* < 0.05). To further investigate the differences, an order-level analysis was performed. As shown in Figure 5B, the MetR diet markedly decreased the relative abundance of *Enterobacteriaceae* and *Enterococcaceae* but increased the relative abundance of *Erysipclotxichaceae* (*p* < 0.05). In addition, the MetR diet significantly decreased the relative abundance of *Escherichia-Shigella* and *Enterococcus* but increased the relative abundance of *Clostridium_sensu_stricto_1* at the genus level (*p* < 0.05) (Figure 5C).

### 2.4. Effects of MetR on Gut Microbiota and Cecal, Colon, and Fecal SCFA Levels 

**Figure 6:**
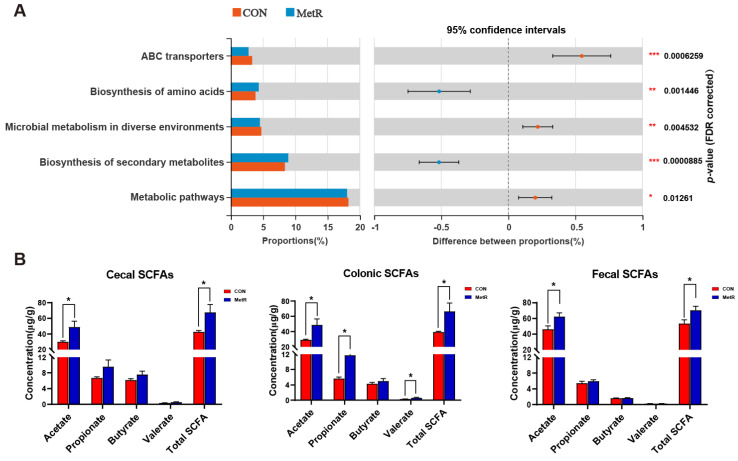
(**A**) Inferred metabolic functions of cecum microbiota by PICRUSt analysis among the two groups. (**B**) Concentrations of SCFAs in cecal content, colon content and fecal content. Data are expressed as the mean ± SEM for *n* = 10, * *p* < 0.05, ** *p* < 0.01, *** *p* < 0.001 versus CON.

The abundances of Kyoto Encyclopedia of Genes and Genome (KEGG) pathways in each group were predicted using PICRUSt (*p* < 0.01, Figure 6A). Notably, the biosynthesis of amino acids and secondary metabolites was significantly enriched in tissues from rats in the MetR group relative to rats in the CON group (Figure 6A). In addition, other metabolic pathways, such as ABC transporters and microbial metabolism in different environments, were significantly upregulated in the CON group but downregulated in the MetR group. Figure 6B shows the levels of SCFAs in cecal, colonic, and fecal contents. The concentrations of acetate and total SCFA were significantly higher in the MetR group than in the CON group (*p* < 0.05). In particular, the concentrations of propionate and valerate in the colonic contents showed a notable increase in the MetR group compared to the CON group (*p* < 0.05, Figure 6B).

### 2.5. Effects of MetR on Plasma Metabolome

**Figure 7:**
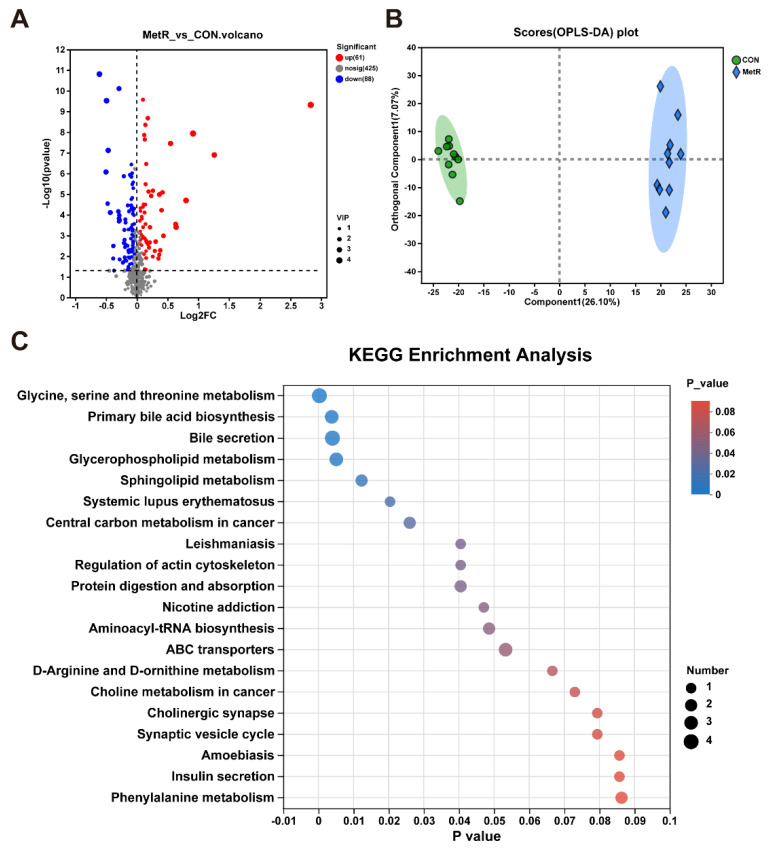
(**A**) Volcano plot for the CON and MetR groups, where each point represents a metabolite. Differentially expressed metabolites in combined CON and MetR (|log2 (Fold Change)| > 1 and nominal *p* < 0.05). Red dots represent upregulated metabolites; blue dots represent downregulated metabolites; and gray dots indicate non-significant differences. (**B**) Scatter plot of scores from OPLS-DA of the CON and MetR groups. (**C**) KEGG pathways enriched in the MetR group compared to the CON group.

**Figure 8:**
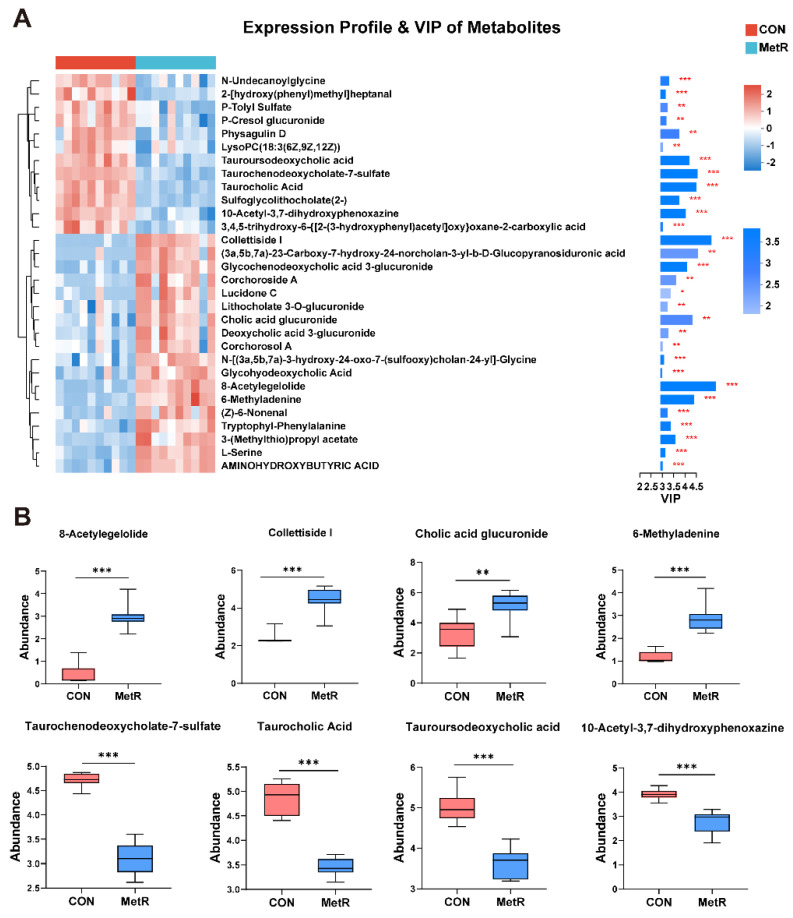
(**A**) Heatmap showing normalized values of 30 metabolites that were differentially abundant among the CON and MetR groups. The normalized abundance values are depicted from red to green, where red represents the highest abundance and green represents the lowest abundance. The dendrogram shows the distances of the metabolites based on their relative abundances. (**B**) The identified and change trend of the potential biomarkers of rat intervened by Wilcox test, unpaired. Data are expressed as the mean ± SEM for *n* = 10, * *p* < 0.05, ** *p* < 0.01, *** *p* < 0.001 versus CON.

A total of 149 metabolites were identified in the rat plasma, among which 61 were upregulated and 88 were downregulated (Figure 7A). A supervised OPLS-DA model (R2 = 0.996, Q2 = 0.916, *p* < 0.05) showed a robust separation of the diet groups as visualized by the score plot (Figure 7B). The KEGG databases were used to establish the metabolic pathways, which included glycine, serine, and threonine metabolism, primary bile acid biosynthesis, and bile secretion (Figure 7C). A total of 30 metabolites had VIP > 1, suggesting that they contribute to the discrimination of the groups (Figure 8A). The levels of 8-acetylegelolide, collettiside I, cholic acid glucuronide, 6-methyladenine, and cholic acid glucuronide were significantly increased in the MetR group relative to the CON group (*p* < 0.01, Figure 8B). In addition, the levels of taurochenodeoxycholate-7-sulfate, taurocholic acid (TCA), tauro-ursodeoxycholic acid (TUDCA), and 10-acetyl-3,7-dihydroxyphenoxazine were markedly reduced in the MetR group relative to the CON group (*p* < 0.01, Figure 8B).

### 2.6. Spearman’s Correlation Analysis between Plasma Metabolites, Colonic SCFAs, and Gut Microbiota

**Figure 9:**
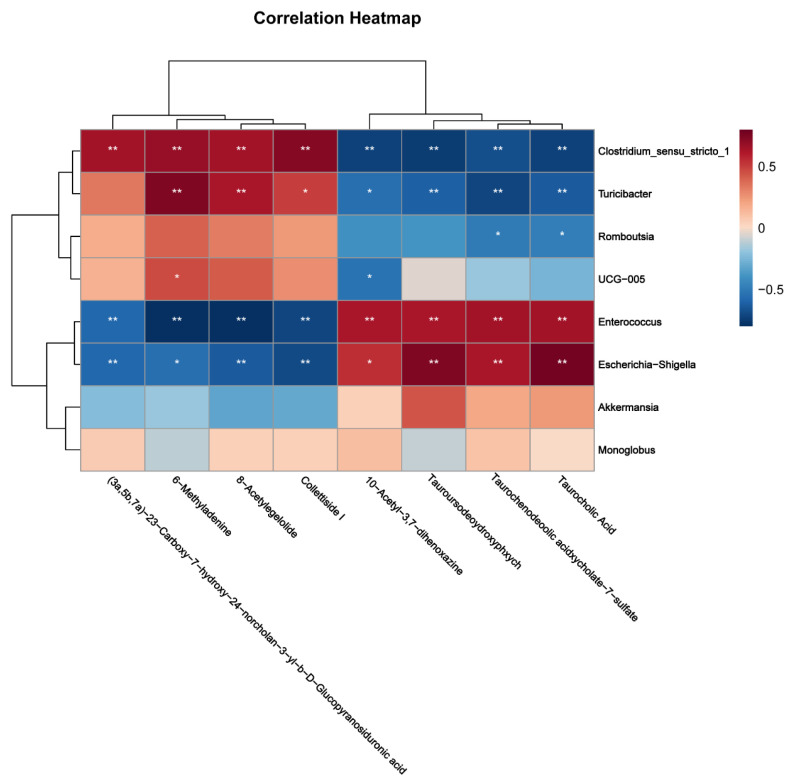
Heat map of the relationship between plasma metabolites, colonic SCFAs and gut microbiota. Red squares represent significant positive correlations (R > 0.5, *p* < 0.05), blue squares represent significant negative correlations (R < −0.5, *p* < 0.05), and white squares represent non-significant correlations. * *p* < 0.05, ** *p* < 0.01.

The correlations between plasma metabolites, colonic SCFAs and gut microbiota were evaluated by Spearman’s correlation analysis (Figure 9). The levels of colonic acetate, total colonic SCFA, 8-acetylegelolide, collettiside I, 6-methyladenine, and cholic acid glucuronide were positively correlated with the abundance of *Clostridium_sensu_stricto_1* but negatively correlated with the abundance of *Escherichia-Shigella* and *Enterococcus*. Furthermore, the levels of taurochenodeoxycholate-7-sulfate, TCA, TUDCA, and 10-acetyl-3,7-dihydroxyphenoxazine were positively correlated with the abundance of *Escherichia-Shigella* and *Enterococcus* but positively correlated with the abundance of *Clostridium_sensu_stricto_1* and *Turicibacter*.

## 3. Discussion

There is overwhelming evidence consistently demonstrating that the MetR diet has many beneficial health effects, including reducing hepatic steatosis [6], ameliorating fat accumulation [16], preventing systemic inflammation [17], and regulating energy and protein homeostasis [7] in high-fat-fed mice. Dietary MetR prevents cognitive impairment in D-galactose-induced aging mice and extends lifespan in progeroid mice [3,18]. In this study, MetR diet increased the abundance of *Clostridium_sensu_stricto_1*, resulting in a considerable upregulation of SCFAs and downregulation of the plasma taurine-conjugated bile acids, like TCA and TUDCA. Moreover, SCFAs facilitated health benefits in the rats, including decreasing systemic inflammation and enhancing the integrity of the gut barrier.

Dietary MetR has been used as a research model for preventing and treating obesity-related metabolic diseases. The gut epithelial barrier is an essential barrier between the environment and the internal milieu of the body, limiting the passage of pathogens and toxins [19]. The disruption of the integrity of the gut barrier allows the passage of bacterial products, leading to the production of pro-inflammatory cytokines [20]. In the current study, we found that MetR reduced the levels of serum and colonic pro-inflammatory factors in rats. This finding may be related to the enhancement of tight junction proteins by MetR. The gut microbiota plays a critical role in gut growth, nutrient absorption, and host health [21]. Higher Simpson index values indicate lower community diversity [22]. The MetR group had the richest microbiota species diversity compared to the CON group, as evidenced by the reduced Simpson indices. Our results indicate that MetR selectively decreased the abundance of *Proteobacteria*. Several studies have shown that *Proteobacteria* are closely correlated with inflammatory bowel disease [23,24]. The integrity of the gut barrier plays a critical role in maintaining gut homeostasis by ensuring a complex crosstalk between gut microbes and the host [25]. Previous studies have shown that pro-inflammatory cytokines facilitate intestinal barrier damage and invasion of pathogens [26]. Ren et al. [27] showed that MetR can improve gut barrier function in aged mice by alleviating the oscillations of inflammation-related microbes. In general, an increase in the relative abundance of *Enterobacteriaceae* causes inflammatory damage to the gut epithelium barrier [28]. Our data showed that the levels of colonic D-lactate, TNF-α, IL-2, IL-6, and IL-12 in the MetR group were significantly lower than those in the CON group. Consistent with this result, MetR decreased the abundance of *Enterobacteriaceae*, *Enterococcaceae*, and *Escherichia-Shigella*; this also corresponded with an increase in *ZO-1*, claudin-3, and claudin-5 mRNA expression [29,30].

SCFAs are important energy sources for intestinal epithelial cells, allowing them to modulate the gut barrier function and host metabolism [12]. The present work revealed that MetR effectively elevated the concentration of SCFAs (acetate, propionate, and valerate) and improved enteral homeostasis. *Erysipclotxichaceae, Bacteroides, Lactobacillus*, and *Clostridium_sensu_stricto_1* consist of microbes that produce SCFAs, which are beneficial for preventing inflammation [31,32]. Moreover, *Proteobacteria* and *Escherichia-Shigella* are associated with microbiota inflammatory properties [33,34]. In this study, we found that MetR selectively increased the abundance of representative SCFAs producers (*Erysipclotxichaceae* and *Clostridium_sensu_stricto_1*), along with enhanced levels of SCFAs in the cecal, colonic, and fecal contents [35,36], indicating that MetR maintained intestinal homeostasis by promoting the growth of beneficial gut bacteria. What is more interesting is that colonic acetate and total SCFAs levels were found to positively correlate with relative *Clostridium_sensu_stricto_1* abundance. This is consistent with a previous study in which MetR increased the abundance of SCFA-producing bacteria, decreased the abundance of inflammation-related bacteria, and improved the intestinal mucosal immune barrier in a time-dependent manner [16]. Furthermore, PICRUSt indicates MetR may have mediated amino acid and secondary metabolite biosynthesis in the gut microbiota. These results suggested that MetR supplementation effectively increased the abundance of SCFA-producing bacteria and increased amino acid biosynthesis in the gut microbiota.

The metabolomic profiling revealed that eight plasma metabolites associated with the MetR diet were mainly involved in glycine–serine–threonine metabolism, primary BA biosynthesis, and bile secretion. This is consistent with the findings of Aon et al. (2020), who reported that glycine–serine–threonine metabolism influences longevity and related life-sustaining mechanisms [37]. Furthermore, BAs promote health and longevity in various organisms [38]. In our study, the levels of taurochenodeoxycholate-7-sulfate, TCA, and TUDCA were reduced and negatively correlated with the abundance of *Clostridium_sensu_stricto_1*. Excess taurine-conjugated BAs in the serum, particularly TCA, TUDCA, and tauro-chenodeoxycholic acid (TCDCA), may exacerbate the progression of non-alcoholic steatohepatitis and the severity of drug-induced liver injury [39,40,41]; this supports our observations in this study, indicating that the reduction in the levels of taurine-conjugated BAs might represent a means by which MetR exerts its hepato-protective effect. Moreover, TCA and TCDCA downregulate the expression of hepatic FXR and are positively associated with pro-inflammatory cytokines, such as TNF-α [42,43], supporting our observation in this study. It has been reported that bile salt hydrolase-producing bacteria such as *Clostridium_sensu_stricto_1* and *Bacteroidetes* hydrolyze taurine- and glycine-conjugated BAs to unconjugated free forms, which can be further converted to secondary BAs [40,44]. Therefore, the increased abundance of *Clostridium_sensu_stricto_1* and *Bacteroidetes* in the cecal contents may account for the depleted taurine-conjugated BAs in the MetR-fed rats.

However, the exact mechanism by which MetR affects serum BAs levels and improves gut health remains to be elucidated.

## 4. Methods and Materials

### 4.1. Animal Experiment

The animal protocols were carried out under the guidelines of animal care and were approved by the Institutional Animal Care and Use Committee of Hunan Agricultural University (approval number: CACAHU 2022-0426). Seven-week-old male Sprague–Dawley rats were purchased from Tianqin Biotechnology (Changsha, China) and were individually housed in a conventional animal facility that was maintained at 22 ± 2 °C and 55 ± 10% relative humidity with a 12-h light/dark photoperiod. After one week of acclimation, all the rats were randomly divided into two experimental diet groups: (1) the control group fed with the normal diet (CON, 0.86% methionine); (2) methionine-restricted diet group (MetR, 0.17% methionine). The composition and nutritional value of the diets are presented in Table S1 as previously described [15,45,46].

### 4.2. Sample Collection and Preparation 

After six weeks of experimental administration, blood samples were collected from the retro-orbital of each rat under fasting conditions and centrifuged at 3500× *g* at 4 °C for 10 min. The separated plasma was then stored in a refrigerator at −80 °C. The contents of the cecum of each rat were removed, snap-frozen in liquid nitrogen, and stored at −80 °C for subsequent 16S rRNA gene sequencing analysis. The contents of the colon and the fresh feces were collected for analysis.

### 4.3. Enzyme-Linked Immunosorbent Assay (ELISA) 

The levels of lipopolysaccharide (LPS) and D-lactate in rat plasma were determined using the ELISA kit (Jiangsu Meimian Industrial Co., Ltd., Yancheng, China). The concentrations of tumor necrosis factor-alpha (TNF-α), interleukin-1 beta (IL-1β), interleukin-2 (IL-2), interleukin-4 (IL-4), interleukin-6 (IL-6), and interleukin-12 (IL-12) were quantified using enzyme-linked immunosorbent assay kits (CSB-E11987r, CSB-E08055r, CSB-E08055r, CSB-E04635r, CSB-E04635r, CSB-E07364r, CUSABIO, Wuhan, China, https://www.cusabio.com/, 4 June 2022) according to the manufacturer’s instructions. The protein concentration of the colon tissues was measured using the Omni-Easy^TM^Instant BCA Protein Assay Kit (ZJ102, Epizyme Biotech; Shanghai; China).

### 4.4. Gene Expression 

Total RNA was isolated from colon tissues using RNAiso Plus (Cat # 9109, Takara Biomedical Technology (Beijing) Co., Ltd., Beijing, China) according to the manufacturer’s instructions. The concentration of each RNA sample was determined using Nanodrop One (Thermo Fisher Scientific, Waltham, MA, USA). Genomic DNA contamination was removed by incubating with the Evo *M-MLV* RT Kit with gDNA Clean for qPCR (AG11705) before reverse transcription. RT-qPCR was conducted using SYBR Green Premix Pro Taq HS qPCR Kit (AG11701, Accurate Biotechnology (Hunan) Co., Ltd., Changsha, China) by real-time PCR instrument (LightCycler480II, Roche, Germany). The relative expression levels of the target genes were calculated using the 2^−ΔΔCt^ method. GAPDH was used as the reference gene.

### 4.5. Histological Analysis

Colon tissues were fixed in 4% paraformaldehyde for 24 h, dehydrated using a gradient of ethanol and xylene, and finally embedded in paraffin. A microtome (Leica, RM2016, Vizna, Germany) was then used to make 4 μm sections that were subsequently dewaxed in xylene. The dewaxed sections were then rehydrated by lowering the concentration of ethanol before being stained with hematoxylin and eosin (H and E). Finally, the sections were dehydrated with gradient ethanol before dehydration with xylene. Carnoy’s colonic tissues were stained with PAS according to the manufacturer’s instructions (Servicebio, Wuhan, China). The sections were briefly stained with PAS dye solution for 10 to 15 min and then rinsed with distilled water. The slides were dehydrated with absolute ethyl alcohol and xylene before image acquisition on an inverted fluorescence microscope (Axio Vert A1, Zeiss, Germany).

### 4.6. Immunofluorescence

Paraffin sections of colon tissue of 4 μm thickness were subjected to antigen extraction with citrate buffer (pH 6.0). Tissue sections were then incubated overnight at 4 °C with primary antibodies (Abcam, UK) against ZO-1 (1:200, ab221547). Next, sections were incubated with Alexa Fluor 488 conjugated secondary antibodies (1:100, Invitrogen, Life Technologies, Carlsbad, CA, USA) for 50 min at 37 °C. Samples were observed under an inverted fluorescence microscope (Axio Vert A1, Zeiss, Germany).

### 4.7. 16S rRNA Gene Sequencing Analysis

Microbial genomic DNA from cecal contents was extracted using the E.Z.N.A.^®^ Soil DNA Kit (Omega Bio-tek, Norcross, GA, USA) according to the manufacturer’s instructions. Total bacterial concentrations in each sample were measured using a qPCR assay that targets the V3–V4 hypervariable region of the 16S rRNA gene from the cecal microbiota was amplified using a forward primer (338F: 5′-ACTCCTACGGGAGGCAGCAG-3′) and a reverse primer (806R: 5′-GGACTACHVGGGTWTCTAAT-3′). The sequencing libraries of bacterial 16S rRNA genes were produced using NEXTFLEX^®^ Rapid DNA-Seq Kit (Bio Scientific, Phoenix, AZ, USA) for high throughput sequencing. The sequencing was carried out at Majorbio Bio-Pharm Technology Co., Ltd. (Shanghai, China) on an Illumina MiSeq PE300 platform. The original DNA fragments were quality filtered and merged using fastp (version 0.20.0) and Flash (version 1.2.11). The sequence reads were classified into operational taxonomic units (OTUs) at 97% identity using Uparse (version 7.1). The RDP classifier (version 2.2) was used to classify and annotate each sequence, and a comparison was conducted against the Silva 16S rRNA database (v138) using a threshold of 70%.

### 4.8. Plasma Metabolomics Analysis

A 50-μL aliquot of plasma in an Eppendorf tube was mixed with a pre-cooled methanol/water solution (4:1, *v*/*v*). The mixture was vortexed for 5 min and centrifuged at 12,000× *g* at 4 °C for 10 min, after which the supernatant was carefully transferred into a new Eppendorf tube and thoroughly dried using a vacuum centrifugal concentrator (SPD130P1, Thermo Fisher, Waltham, MA, USA). A volume of 200 µL of acetonitrile: water (1:1) was added to completely dissolve the dried supernatant. After centrifugation at 12,000× *g* at 4 °C for 5 min, the sample was carefully transferred into sample vials for combined ultra-high liquid chromatography–mass spectrometry (Q-Exactive Plus, Thermo Fisher, USA). The EZinfo 2.0 software was used to perform orthogonal partial least squares discriminant analysis (OPLS-DA) by analyzing each ion. All metabolite variables were scaled using the Pareto method before conducting the OPLS-DA. The model validity was evaluated using model parameters R2 and Q2, which provide information on the interpretability and predictability of the model, respectively, and avoid the risk of over-fitting. Metabolites with variable importance in the projection (VIP) > 1 and *p* < 0.05 were considered statistically significant.

### 4.9. SCFAs

Colonic, cecal, and fecal content samples (1 g) were mixed with ultrapure water via processes of homogenization and centrifugation. The supernatant was collected in a 10 mL Eppendorf tube, mixed with 25% metaphosphate at a ratio of 9:1 (*v*/*v*) and filtered through a 0.22 μm membrane filter. The SCFAs (acetate, propionate, butyrate, and valerate) were quantified using gas chromatography (8890, Agilent, Santa Clara, CA, USA).

### 4.10. Statistical Analysis

Data were analyzed using the IBM SPSS 23.0 statistical software (SPSS Inc., Chicago, IL, USA). Differences between means were tested using Student’s *t*-test. Beta-diversity containing principal co-ordinates analysis (PCoA) with a Bray-Curtis diversity distance matrix were performed using R software (version 3.3.1). The different relative abundance of bacteria within groups was calculated by Wilcoxon rank-sum test. The Spearman correlation coefficients were generated using GraphPad Prism (version 9.5.0). All data are presented as mean ± SEM. *p* < 0.05 was considered statistically significant.

## 5. Conclusions

These results indicated that MetR treatment increased the concentration of intestinal SCFAs by remodeling the gut microbiota, as shown by the increased relative abundance of *Erysipclotxichaceae* and *Clostridium_sensu_stricto_1* and the decreased relative abundance of *Proteobacteria, Enterobacteriaceae*, *Enterococcaceae*, and *Escherichia-Shigella* in the cecum. The results indicate that MetR has the potential to enhance the integrity of the gut barrier and reduce the levels of taurine-conjugated BAs. These alterations in plasma metabolite levels have contributed to the beneficial effects of MetR. Therefore, MetR improved gut health and modified plasma BA levels and conjugation by regulating the gut microbiota in rats. These findings offer valuable insights that can guide the implementation of nutritional strategies aimed at promoting gut health and longevity.

## Data Availability

Data will be made available on request. Sequencing data generated in this study were deposited in Sequence Read Archive (SRA) with the SRA accession numbers PRJNA1065593.

## Supplementary Materials

The following supporting information can be downloaded at: https://www.mdpi.com/article/10.3390/ijms25073657/s1.

## Abbreviations

MetR, Methionine restriction; LPS, lipopolysaccharide; DAO, diamine oxidase; TNF-α, tumor Necrosis Factor Alpha; IL-1β, interleukin-1 Inhibitors; IL-2, interleukin-2; IL-4, interleukin-4; IL-6, interleukin-6; IL-12, interleukin-12; ZO-1, zonula occludens-1; H and E, hematoxylin and eosin; SCFAs, short-chain fatty acids; OPLS-DA, orthogonal partial least squares discriminant analysis; VIP, variable importance in the projection; TCA, taurocholic acid; TUDCA, tauro-ursodeoxycholic acid; TCDCA, tauro-chenodeoxycholic acid; BA, bile acid.

## References

[1] Gao X., Sanderson S.M., Dai Z., Reid M.A., Cooper D.E., Lu M., Richie J.P., Ciccarella A., Calcagnotto A., Mikhael P.G. (2019). Dietary methionine influences therapy in mouse cancer models and alters human metabolism. Nature.

[2] Han L., Wu G., Feng C., Yang Y., Li B., Ge Y., Jiang Y., Shi Y., Le G. (2020). Dietary methionine restriction improves the impairment of cardiac function in middle-aged obese mice. Food Funct..

[3] Bárcena C., Quirós P.M., Durand S., Mayoral P., Rodríguez F., Caravia X.M., Mariño G., Garabaya C., Fernández-García M.T., Kroemer G. (2018). Methionine Restriction Extends Lifespan in Progeroid Mice and Alters Lipid and Bile Acid Metabolism. Cell Rep..

[4] Kitada M., Ogura Y., Monno I., Xu J., Koya D. (2021). Effect of Methionine Restriction on Aging: Its Relationship to Oxidative Stress. Biomedicines.

[5] Mladenović D., Radosavljević T., Hrnčić D., Rasic-Markovic A., Stanojlović O. (2019). The effects of dietary methionine restriction on the function and metabolic reprogramming in the liver and brain—Implications for longevity. Rev. Neurosci..

[6] Yang Y., Wang Y., Sun J., Zhang J., Guo H., Shi Y., Cheng X., Tang X., Le G. (2019). Dietary methionine restriction reduces hepatic steatosis and oxidative stress in high-fat-fed mice by promoting H_2_S production. Food Funct..

[7] Yang Y., Zhang J., Wu G., Sun J., Wang Y., Guo H., Shi Y., Cheng X., Tang X., Le G. (2018). Dietary methionine restriction regulated energy and protein homeostasis by improving thyroid function in high fat diet mice. Food Funct..

[8] Gasaly N., de Vos P., Hermoso M.A. (2021). Impact of Bacterial Metabolites on Gut Barrier Function and Host Immunity: A Focus on Bacterial Metabolism and Its Relevance for Intestinal Inflammation. Front. Immunol..

[9] Martel J., Chang S.H., Ko Y.F., Hwang T.L., Young J.D., Ojcius D.M. (2022). Gut barrier disruption and chronic disease. Trends Endocrinol. Metab. TEM.

[10] Banerjee S., Sindberg G., Wang F., Meng J., Sharma U., Zhang L., Dauer P., Chen C., Dalluge J., Johnson T. (2016). Opioid-induced gut microbial disruption and bile dysregulation leads to gut barrier compromise and sustained systemic inflammation. Mucosal Immunol..

[11] Schirmer M., Smeekens S.P., Vlamakis H., Jaeger M., Oosting M., Franzosa E.A., Ter Horst R., Jansen T., Jacobs L., Bonder M.J. (2016). Linking the Human Gut Microbiome to Inflammatory Cytokine Production Capacity. Cell.

[12] Martin-Gallausiaux C., Marinelli L., Blottière H.M., Larraufie P., Lapaque N. (2021). SCFA: Mechanisms and functional importance in the gut. Proc. Nutr. Soc..

[13] Akhtar M., Chen Y., Ma Z., Zhang X., Shi D., Khan J.A., Liu H. (2022). Gut microbiota-derived short chain fatty acids are potential mediators in gut inflammation. Anim. Nutr. (Zhongguo Xu Mu Shou Yi Xue Hui).

[14] Ma J., Hong Y., Zheng N., Xie G., Lyu Y., Gu Y., Xi C., Chen L., Wu G., Li Y. (2020). Gut microbiota remodeling reverses aging-associated inflammation and dysregulation of systemic bile acid homeostasis in mice sex-specifically. Gut Microbes.

[15] Ramalingam A., Wang X., Gabello M., Valenzano M.C., Soler A.P., Ko A., Morin P.J., Mullin J.M. (2010). Dietary methionine restriction improves colon tight junction barrier function and alters claudin expression pattern. Am. J. Physiol. Cell Physiol..

[16] Wu G., Shi Y., Han L., Feng C., Ge Y., Yu Y., Tang X., Cheng X., Sun J., Le G.W. (2020). Dietary Methionine Restriction Ameliorated Fat Accumulation, Systemic Inflammation, and Increased Energy Metabolism by Altering Gut Microbiota in Middle-Aged Mice Administered Different Fat Diets. J. Agric. Food Chem..

[17] Yang Y., Zhang Y., Xu Y., Luo T., Ge Y., Jiang Y., Shi Y., Sun J., Le G. (2019). Dietary methionine restriction improves the gut microbiota and reduces intestinal permeability and inflammation in high-fat-fed mice. Food Funct..

[18] Xu Y., Yang Y., Li B., Xie Y., Shi Y., Le G. (2022). Dietary methionine restriction improves gut microbiota composition and prevents cognitive impairment in D-galactose-induced aging mice. Food Funct..

[19] Barbara G., Barbaro M.R., Fuschi D., Palombo M., Falangone F., Cremon C., Marasco G., Stanghellini V. (2021). Inflammatory and Microbiota-Related Regulation of the Intestinal Epithelial Barrier. Front. Nutr..

[20] Bischoff S.C., Barbara G., Buurman W., Ockhuizen T., Schulzke J.D., Serino M., Tilg H., Watson A., Wells J.M. (2014). Intestinal permeability--a new target for disease prevention and therapy. BMC Gastroenterol..

[21] Kuziel G.A., Rakoff-Nahoum S. (2022). The gut microbiome. Curr. Biol. CB.

[22] Xue Y., Jin T., Gao C., Li C., Zhou T., Wan D., Yang M. (2022). Effects of biodegradable film mulching on bacterial diversity in soils. Arch. Microbiol..

[23] Zhao C., Hu X., Bao L., Wu K., Zhao Y., Xiang K., Li S., Wang Y., Qiu M., Feng L. (2022). Gut dysbiosis induces the development of mastitis through a reduction in host anti-inflammatory enzyme activity by endotoxemia. Microbiome.

[24] Yang M., Gu Y., Li L., Liu T., Song X., Sun Y., Cao X., Wang B., Jiang K., Cao H. (2021). Bile Acid-Gut Microbiota Axis in Inflammatory Bowel Disease: From Bench to Bedside. Nutrients.

[25] Stolfi C., Maresca C., Monteleone G., Laudisi F. (2022). Implication of Intestinal Barrier Dysfunction in Gut Dysbiosis and Diseases. Biomedicines.

[26] Kaminsky L.W., Al-Sadi R., Ma T.Y. (2021). IL-1β and the Intestinal Epithelial Tight Junction Barrier. Front. Immunol..

[27] Ren B., Wang L., Mulati A., Liu Y., Liu Z., Liu X. (2021). Methionine Restriction Improves Gut Barrier Function by Reshaping Diurnal Rhythms of Inflammation-Related Microbes in Aged Mice. Front. Nutr..

[28] Zhai Z., Zhang F., Cao R., Ni X., Xin Z., Deng J., Wu G., Ren W., Yin Y., Deng B. (2019). Cecropin A Alleviates Inflammation Through Modulating the Gut Microbiota of C57BL/6 Mice With DSS-Induced IBD. Front. Microbiol..

[29] Jang H.M., Kim J.K., Joo M.K., Shin Y.J., Lee K.E., Lee C.K., Kim H.J., Kim D.H. (2022). Enterococcus faecium and Pediococcus acidilactici deteriorate Enterobacteriaceae-induced depression and colitis in mice. Sci. Rep..

[30] Li S., Guo J., Liu R., Zhang F., Wen S., Liu Y., Ren W., Zhang X., Shang Y., Gao M. (2022). Predominance of Escherichia-Shigella in Gut Microbiome and Its Potential Correlation with Elevated Level of Plasma Tumor Necrosis Factor Alpha in Patients with Tuberculous Meningitis. Microbiol. Spectr..

[31] Liu S., Li E., Sun Z., Fu D., Duan G., Jiang M., Yu Y., Mei L., Yang P., Tang Y. (2019). Altered gut microbiota and short chain fatty acids in Chinese children with autism spectrum disorder. Sci. Rep..

[32] Gao X., Du L., Randell E., Zhang H., Li K., Li D. (2021). Effect of different phosphatidylcholines on high fat diet-induced insulin resistance in mice. Food Funct..

[33] Baltazar-Díaz T.A., González-Hernández L.A., Aldana-Ledesma J.M., Peña-Rodríguez M., Vega-Magaña A.N., Zepeda-Morales A.S.M., López-Roa R.I., Del Toro-Arreola S., Martínez-López E., Salazar-Montes A.M. (2022). Escherichia/Shigella, SCFAs, and Metabolic Pathways-The Triad That Orchestrates Intestinal Dysbiosis in Patients with Decompensated Alcoholic Cirrhosis from Western Mexico. Microorganisms.

[34] Han L., Li T., Du M., Chang R., Zhan B., Mao X. (2019). Beneficial Effects of Potentilla discolor Bunge Water Extract on Inflammatory Cytokines Release and Gut Microbiota in High-Fat Diet and Streptozotocin-Induced Type 2 Diabetic Mice. Nutrients.

[35] Firrman J., Liu L., Mahalak K., Tanes C., Bittinger K., Tu V., Bobokalonov J., Mattei L., Zhang H., Van den Abbeele P. (2022). The impact of environmental pH on the gut microbiota community structure and short chain fatty acid production. FEMS Microbiol. Ecol..

[36] Soriano-Lerma A., García-Burgos M., Alférez M.J.M., Pérez-Carrasco V., Sanchez-Martin V., Linde-Rodríguez Á., Ortiz-González M., Soriano M., García-Salcedo J.A., López-Aliaga I. (2022). Gut microbiome-short-chain fatty acids interplay in the context of iron deficiency anaemia. Eur. J. Nutr..

[37] Aon M.A., Bernier M., Mitchell S.J., Di Germanio C., Mattison J.A., Ehrlich M.R., Colman R.J., Anderson R.M., de Cabo R. (2020). Untangling Determinants of Enhanced Health and Lifespan through a Multi-omics Approach in Mice. Cell Metab..

[38] Bárcena C., Valdés-Mas R., Mayoral P., Garabaya C., Durand S., Rodríguez F., Fernández-García M.T., Salazar N., Nogacka A.M., Garatachea N. (2019). Healthspan and lifespan extension by fecal microbiota transplantation into progeroid mice. Nat. Med..

[39] Li X., Zhao W., Xiao M., Yu L., Chen Q., Hu X., Zhao Y., Xiong L., Chen X., Wang X. (2022). Penthorum chinense Pursh. extract attenuates non-alcholic fatty liver disease by regulating gut microbiota and bile acid metabolism in mice. J. Ethnopharmacol..

[40] Zhang X., Coker O.O., Chu E.S., Fu K., Lau H.C.H., Wang Y.X., Chan A.W.H., Wei H., Yang X., Sung J.J.Y. (2021). Dietary cholesterol drives fatty liver-associated liver cancer by modulating gut microbiota and metabolites. Gut.

[41] Tian Q., Yang R., Wang Y., Liu J., Wee A., Saxena R., Wang L., Li M., Liu L., Shan S. (2021). A High Serum Level of Taurocholic Acid Is Correlated With the Severity and Resolution of Drug-induced Liver Injury. Clin. Gastroenterol. Hepatol. Off. Clin. Pract. J. Am. Gastroenterol. Assoc..

[42] Jia W., Xie G., Jia W. (2018). Bile acid-microbiota crosstalk in gastrointestinal inflammation and carcinogenesis. Nat. Rev. Gastroenterol. Hepatol..

[43] Wang L., Ren B., Zhang Q., Chu C., Zhao Z., Wu J., Zhao W., Liu Z., Liu X. (2020). Methionine restriction alleviates high-fat diet-induced obesity: Involvement of diurnal metabolism of lipids and bile acids. Biochim. Biophys. Acta. Mol. Basis Dis..

[44] Shen B., Zhou C., Gu T., Shen Z., Guo Y., Dai W., Liu Y., Zhang J., Lu L., Dong H. (2022). Kuhuang alleviates liver fibrosis by modulating gut microbiota-mediated hepatic IFN signaling and bile acid synthesis. Front. Pharmacol..

[45] Maddineni S., Nichenametla S., Sinha R., Wilson R.P., Richie J.P. (2013). Methionine restriction affects oxidative stress and glutathione-related redox pathways in the rat. Exp. Biol. Med..

[46] Tamanna N., Kroeker K., Braun K., Banh S., Treberg J.R. (2019). The effect of short-term methionine restriction on glutathione synthetic capacity and antioxidant responses at the whole tissue and mitochondrial level in the rat liver. Exp. Gerontol..

