# Supplementary material for: Dietary Methionine Restriction Improves Gut Health and Alters the Plasma Metabolomic Profile in Rats by Modulating the Composition of the Gut Microbiota
Source: Int J Mol Sci. 2024 Mar 25;25(7):3657. doi: 10.3390/ijms25073657 (PMC11011829; doi:10.3390/ijms25073657)
Supplement: Supplementary file 1 [file ijms-25-03657-s001.zip › ijms-2923499-supplementary.pdf]

Table S1. Composition of the methionine-restricted and control diets.

Ingredient (%)	CON	MetR
L-Arginine	1.12	1.12
L-Lysine	1.44	1.44
L-Histidine	0.33	0.33
L-Leucine	1.11	1.11
L-Isoleucine	0.82	0.82
L-Valine	0.82	0.82
L-Threonine	0.82	0.82
L-Tryptophan	0.18	0.18
L-Methionine	0.86	0.17
Glutamic acid	2.70	3.39
L-Phenylalanine	1.16	1.16
Glycine	2.33	2.33
Dextrin	5.00	5.00
Corn starch	43.61	43.61
Sucrose	20.0	20.0
Solca floc	5.00	5.00
Choline bitartrate	0.20	0.20
Vitamin mix-AIN	1.00	1.00
Mineral mix-AIN	3.50	3.50
Corn oil	8.00	8.00
Total	100.00	100.00
